# Visual detection of porcine epidemic diarrhea virus using a novel reverse transcription polymerase spiral reaction method

**DOI:** 10.1186/s12917-019-1851-7

**Published:** 2019-04-15

**Authors:** Xueyu Wang, Xin Xu, Wen Hu, Kejing Zuo, Zhili Li, Yunchao Kan, Lunguang Yao, Jun Ji, Yingzuo Bi

**Affiliations:** 1 Henan Provincial Engineering Laboratory of Insects Bio-reactor, China-UK-NYNU-RRes Joint Laboratory of Insect Biology, Nanyang Normal University, Wolong Road 1638, Nanyang, 473061 People’s Republic of China; 2 Veterinary Laboratory, Guangzhou Zoo, Guangzhou, 510642 People’s Republic of China; 3 College of Animal Science, South China Agricultural University, Guangzhou, 510642 People’s Republic of China

**Keywords:** Reverse transcription polymerase spiral reaction (RT-PSR), Porcine epidemic diarrhea virus (PEDV), Sensitivity, Specificity; detection

## Abstract

**Background:**

Porcine epidemic diarrhea virus (PEDV) is a major etiological agent of porcine epidemic diarrhea around the world. Point-of-care testing in the field is lacking owing to the requirement for a simple, robust field applicable test that does not require professional laboratory equipment. The aim of this study was to establish a novel reverse transcription polymerase spiral reaction (RT-PSR) assay for the rapid detection of porcine epidemic diarrhea virus (PEDV). For the assay, a specific RT-PSR primer pair was designed against a conserved region in PEDV ORF3.

**Results:**

The RT-PSR was optimized, and PEDV could be detected after a 50 min incubation at 62 °C, in addition to the 15 min required for reverse transcription. No cross-reaction with other porcine infectious viruses was observed. This new method for PEDV detection was 10 times more sensitive than the conventional reverse transcription-polymerase chain reaction (RT-PCR) assay. The positive rates for 65 clinical samples using the new RT-PSR assay and the conventional RT-PCR assay were 58.46% (38/65) and 53.84% (35/65), respectively. In the RT-PSR assay, the addition of a mixture of dyes allowed a positive reaction to be directly observed by the naked eye.

**Conclusions:**

These results indicate that this RT-PSR assay is capable of accurately detecting PEDV, and has the advantages of high specificity and sensitivity for the detection of PEDV.

## Background

Porcine epidemic diarrhea (PED) is a highly contagious swine enteritis accompanied by vomiting, watery diarrhea, dehydration, and other symptoms [1]. PED is caused by the porcine epidemic diarrhea virus (PEDV), which belongs to the order *Nidovirales* and the family *Coronaviridae* [2]. PEDV infections are more frequent in the winter [3], and although PEDV can infect swine of all ages, it causes the most serious harm to suckling piglets. Since the discovery of PED in England in 1971, the disease has expanded to many countries in Europe and Asia, especially China and South Korea, which has caused huge economic losses [4]. In China, PEDV is widely distributed, and outbreaks have a strong negative impact on the swine industry, in part due to the acute onset and fast spread of the virus,the incidence rate in piglets can be very prevalent [5–7].

The methods currently used to diagnose PED in clinical samples are mainly divided into two types: 1) immunological methods including immunochromatography and enzyme-linked immunosorbent assay (ELISA) [8, 9], and 2) molecular biology assays including conventional reverse transcription-polymerase chain reaction (RT-PCR), reverse transcription quantitative PCR (RT-qPCR), multiplex PCR, and reverse transcription loop-mediated isothermal amplification (RT-LAMP) [10–13]. Although immunological methods are generally low cost and easy to perform, they have several disadvantages, including inconclusive results and the long time required to perform the assays. To decrease the time required for PEDV detection, PCR-related methods focused on the amplification of viral nucleic acids have been developed, which have been shown to be more efficient, highly sensitive and specific, even at different stages of the disease, when compared to immunological diagnostic methods. However, these molecular diagnostic methods cannot be widely used because of their complex operation, time-consuming nature, and the requirement for expensive instrumentation.

Detection methods based on isothermal amplification of nucleic acids, which can rapidly synthesize large amounts of DNA without any specific requirements for precision instruments, have been widely used. The polymerase spiral reaction (PSR) [14] is a novel nucleic acid isothermal amplification method that has the advantages of simplicity, rapidity, accuracy, and low cost when compared to conventional PCR. In addition, less primer is required, and primer design is simpler than for loop-mediated isothermal amplification (LAMP). Due to the high efficiency of amplification, product formation is accompanied by high levels of pyrophosphate ion by-product, leading to a change in pH. Therefore, a pH-sensitive dye can be used to detect the product of the reaction with the naked eye [15]. PSR detection methods have been used for numerous human and veterinary pathogens [16–19]. Therefore, we evaluated clinical samples using the newly developed RT-PSR method to determine the method’s utility for early detection of PEDV.

## Results

### Optimum reaction temperature and time for the diagnosis of PEDV by RT-PSR

Electrophoretograms showed no obvious difference in the gradient bands produced at temperatures ranging from 60 °C to 64 °C; however, the bands were slightly more obvious at 62 °C. With increasing reaction time, the bands become more visible, and reached a peak at 50 min. Therefore, the optimum temperature and time for detecting PEDV by RT-PSR was 62 °C and 50 min, respectively. The RT-PSR assay for PEDV detection was optimized as follows: reverse transcription at 42 °C for 15 min and spiral amplification at 62 °C for 50 min.

### Sensitivity of the RT-PSR assay

**Fig. 1:**
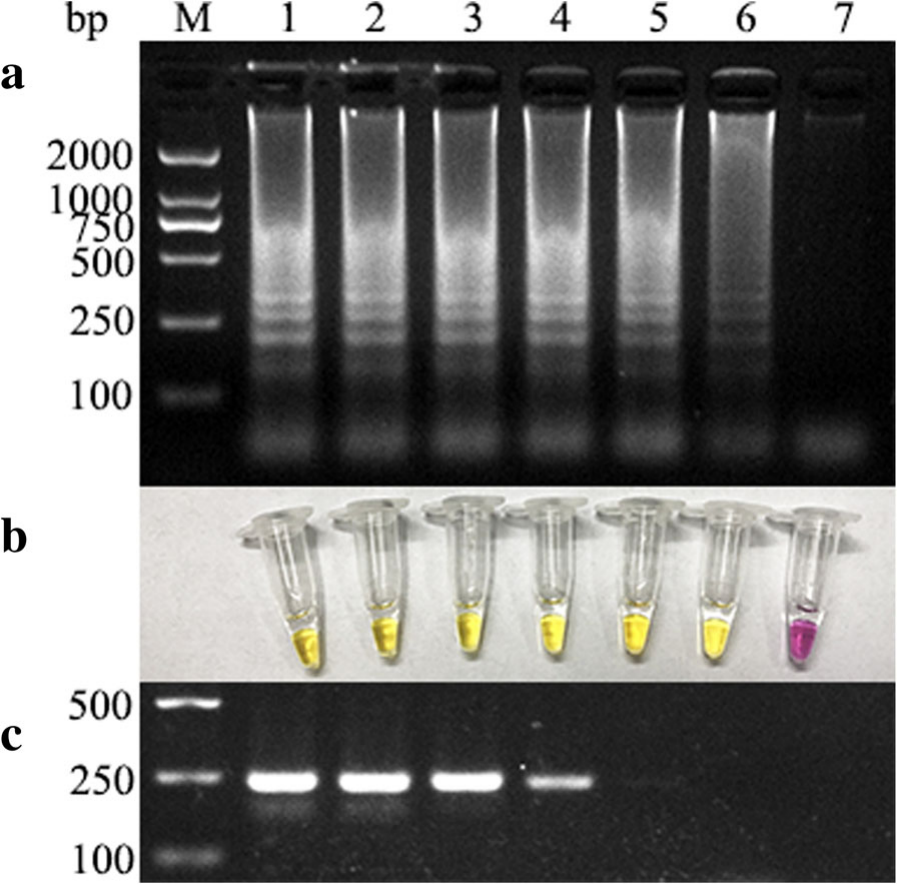
Sensitivity of the RT-PSR and RT-PCR assays for the detection of PEDV. Ten-fold serial dilutions of PEDV RNA were subjected to the RT-PSR and RT-PCR assays and analyzed. Lane M, bases pair (bp) marker DL2000. Lanes 1–7, dilutions of PEDV RNA (10^0^, 10^− 1^, 10^− 2^, 10^− 3^, 10^− 4^, 10^− 5^, and 10^− 6^). **a** Agarose gel electrophoresis demonstrating the sensitivity of the RT-PSR assay. **b** Colorimetric analysis demonstrating the sensitivity of the RT-PSR assay. **c** Agarose gel electrophoresis demonstrating the sensitivity of the RT-PCR assay

Samples were serially diluted tenfold (10^− 1^, 10^− 2^, 10^− 3^, 10^− 4^, 10^− 5^, and 10^− 6^) and were used in both the RT-PSR and conventional PCR assays for PEDV detection, and the results are shown in Fig. 1 At the 10^− 4^ dilution, conventional PCR yielded the clear bands. However, at dilutions below 10^− 4^, there was no obvious band (Fig. 1c). The results in Fig. 1a demonstrated that when the concentration was 10^− 5^, the PSR produced a clear ladder banding pattern; however, there were no obvious bands when the concentration was below 10^− 5^. Therefore, the RT-PSR assay is more sensitive than the conventional RT-PCR assay. Based on the results of this experiment, the RT-PSR method can be used to detect PEDV, and with the addition of a colorimetric dye, positive clinical samples containing amplified product were orange-yellow, while negative samples were purple under natural light. Thus, confirmation of a positive result can be visually confirmed with the naked eye (Fig. 1b).

### Determination of RT-PSR specificity

**Fig. 2:**
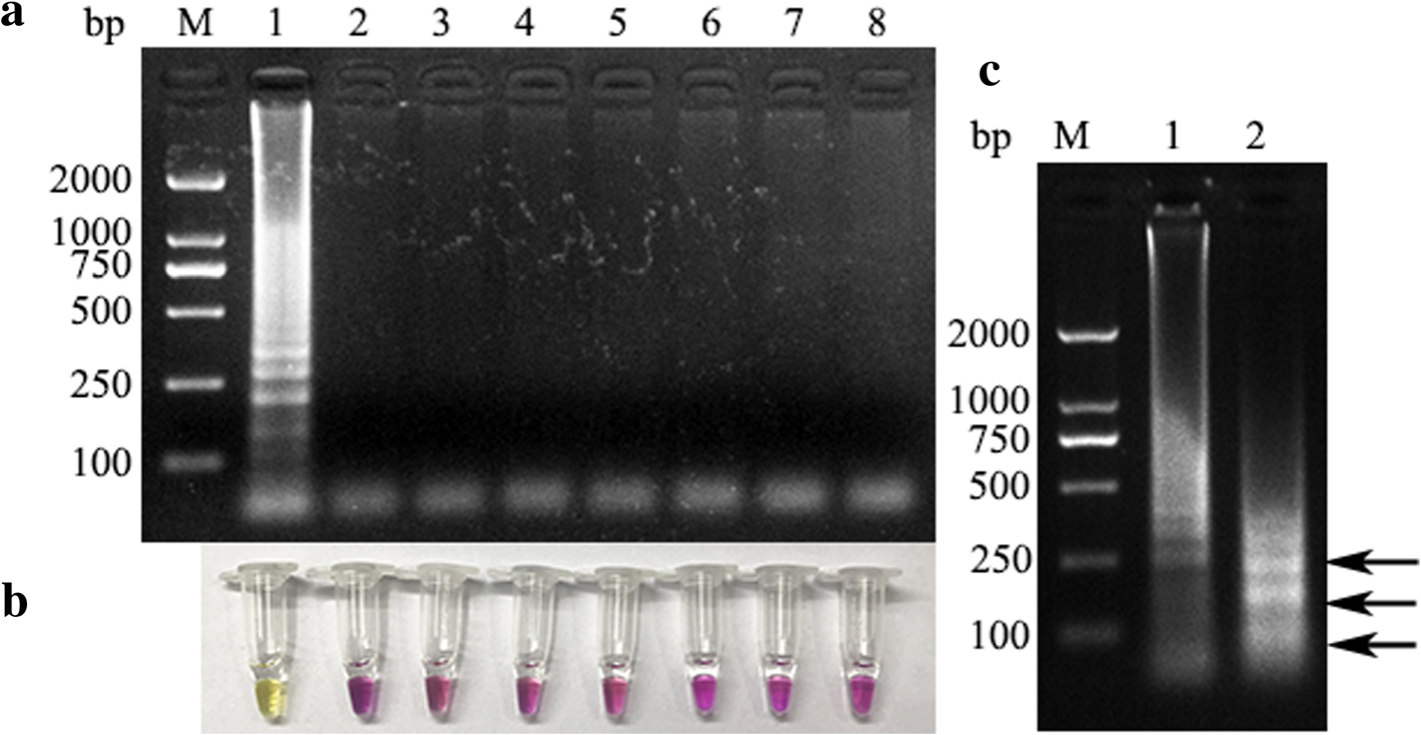
Determination of RT-PSR specificity. Lane M, bases pair (bp) marker DL2000; lane 1, PEDV; lane 2, CSFV; lane 3, PRRSV; lane 4, TGEV; lane 5, PRV; lane 6, PCV2; lane 7, PPV; and lane 8, negative control. **a** Specificity of the RT-PSR assay as determined by electrophoretic separation of the reaction products. **b** Colorimetric analysis of the reactions demonstrating the specificity of the RT-PSR assay. **c** Identification of the RT-PSR products by enzyme digestion. Lane M, molecular size marker DL2000; Lane 1, RT-PSR products; Lane 2: *Eco*R I-digested RT-PSR products

The specificity of the RT-PSR method was tested using selected reference swine viruses. Figure 2a shows that only the reaction containing PEDV yielded an obvious ladder, which demonstrates that the RT-PSR assay specifically detected PEDV, and no other tested viral pathogen was detected. Figure 2b shows the corresponding reactions containing a pH indicator dye.

The product was digested with *Eco*R I, and the results are shown in Fig. 2c. Based on the primer design, digestion of the amplified PEDV product with *Eco*R I should yield three main bands of 220, 182, and 42 bp. The results of the experiment are in good agreement with these theoretical values, thus verifying the identity of the amplification products. This further confirms that the RT-PSR method is highly accurate for PEDV detection at the molecular level.

### Clinical sample testing

The results of the RT-PSR and conventional PCR assays for clinical isolates detection are shown in Table 1, the positive rates for the RT-PSR and conventional RT-PCR methods were 58.46% (38/65) and 53.84% (35/65), respectively. The RT-PCR-negative samples were confirmed the presence of PEDV by virus isolation. The RT-PSR and RT-PCR assay for detecting PEDV possessed an analytical specificity of 100% (0 false negative) and 92.1% (3 false negative), respectively. These results demonstrate that conventional PCR is not adequately sensitive to detect PEDV in samples with low viral loads or the PSR assay is less affected by potential inhibitors within the samples. Moreover, the efficiency of the developed detection method is not affected by co-infection. Based on the above results, the accuracy of the RT-PSR method is well suited for the detection of early viral infections.

**Table 1:** Comparison of the results generated by the RT-PSR and RT-PCR assays for PEDV using clinical samples

Province	Number of samples	Date	Number of positive samples			Other positive tests
			Virus isolation	RT-PSR	RT-PCR	
Jiangxi	7	2017.03	5	5	4	PoRV
Jiangsu	8	2017.10	3	3	3	TGEV, PRRSV
Jiangsu	7	2017.11	3	3	2	–
Hunan	9	2018.03	5	5	5	PRRSV
Hubei	6	2018.03	3	3	3	TGEV, PCV2
Anhui	9	2018.04	6	6	6	TGEV
Hubei	7	2018.04	4	4	4	PBoV
Anhui	5	2018.05	5	5	5	PRV
Jiangsu	7	2018.09	4	4	3	–
Total	65		38	38	35	

## Discussion

PEDV, a predominant cause of acute enteric infection in swine, leads to severe dehydrating diarrhea and economic losses in the swine industry worldwide. In recent years, the incidence rates of various infectious diseases in swine have been rapidly increasing, and co-infection with PEDV and a variety of other porcine diseases is becoming more and more common [20]. Moreover, the cure rate for PEDV is significantly higher in the early stage of infection than in the late stage of infection. Therefore, it is necessary to establish a rapid, sensitive, and specific method for detecting PEDV early in primary veterinary clinics, which would be a major breakthrough for this disease.

At present, numerous established laboratory diagnostic techniques are used to identify PEDV. Kim [21] compared PEDV detection using RT-PCR, immunohistochemistry, and in situ hybridization; Zhou [22] developed and evaluated three assays, including conventional RT-PCR, SYBR Green I real-time RT-PCR, and TaqMan real-time RT-PCR assays. Their results indicated that the TaqMan real-time RT-PCR could be a useful tool for clinical diagnosis, epidemiological surveys, and outbreak investigations of PEDV. In addition, although RT-PCR detected PEDV more frequently than serological techniques, when only tissues are submitted, immunohistochemistry and in situ hybridization would be useful methods for the detection of PEDV antigen and nucleic acids. A comprehensive analysis showed that although immunological techniques can rapidly and quantitatively detect PEDV in samples, molecular technologies have become more popular for their superior specificity and sensitivity. By combining current research and technological progress, we developed a more suitable molecular-based assay for the detection of PEDV for early diagnosis.

Because of the difficulties in clinical diagnosis, the low accuracy of serological diagnosis, and the complicated operation of diagnosis via viral isolation methods, PSR technology has been widely used for the differential diagnosis of animal epidemics, such as canine parvovirus 2 (CPV-2) [18], African swine fever virus (ASFV) [19], and bovine herpesvirus 1 (BHV-1) [23]. PSR not only has the advantages of traditional PCR, as it can also detect pathogenic genes efficiently and specifically, it also has a shorter detection time, is easier to perform, and uses less reagents, when compared to traditional PCR. Specific nucleic acids can be amplified by the PSR method under isothermal conditions, without the need for template pre-denaturation, or sacrificing amplification efficiency. In a positive reaction, the large amount of products, and white pyrophosphoric acid precipitates make a positive result easy to detect. In this experiment, by using the PEDV ORF3 sequence in GenBank, a highly conserved region of the sequence was analyzed and selected for primer design, and a fast, simple, and accurate PEDV RT-PSR method was successfully developed for the detection of PEDV. It offers many advantages compared to conventional PCR, and the most obvious advantage of this assay is that it is sensitive for PEDV. RT-PSR is more accurate than RT-PCR for the detection of clinical samples, and its sensitivity is about 10 times higher than that of conventional PCR. Further analysis of the RT-PSR-positive samples that were not detected by RT-PCR by viral isolation and identification confirmed the presence of PEDV. We speculated that, for such samples, the viral load may be low or it may have been collected early in the course of the infection. Besides limiting by viral load, PSR assay is less affected by potential inhibitors within the samples, which indicated that RNA Extraction of from samples could be omitted [24]. So far, extraction of nucleic acid from clinical samples was consumedly increased the time and cost for diagnosis on farm or in typical veterinary clinics. Therefore, nucleic acid adsorption or other simple strategy needed study and evaluate for application of RT-PSR assay. In addition, by using the developed RT-PSR detection method, PEDV can be amplified across a wide range of temperatures from 60 °C to 65 °C; therefore, it can be performed in a water bath. In addition, expensive electrophoresis equipment and gel imaging systems are required to analyze the results of conventional PCR, which increases the detection costs. In contrast, using the PSR method established in this study, a positive result can be directly determined by the naked eye when mixed dyes are added before the reaction, which do not affect the amplification efficiency, nor agarose gel electrophoresis. These advantages facilitate the rapid detection of PEDV.

## Conclusions

Through the aforementioned experiments and analyses, we concluded that the developed RT-PSR method offers multiple advantages compared to conventional PCR, including shorter time and higher sensitivity. The popularization of this technology for PEDV detection will be an important development in the study of PED in China, which should be beneficial for the prevention and treatment of PEDV in the swine industry.

## Methods

### Viruses and clinical samples

Tissue samples were collected from pigs died from diarrhea symptoms on farms, the small intestine was immersed in phosphate-buffered saline (PBS), washed several times to eliminate residual blood, and then placed in a centrifuge tube and stored at − 80 °C until use.

The experimental procedure for virus isolation is in African green monkey kidney (VERO) cells according to reference [25]. The isolates were determined by more propagation until apparent cytopathic effects appeared.

Classical swine fever virus (CSFV), porcine reproductive and respiratory syndrome virus (PRRSV), transmissible gastroenteritis virus (TGEV), porcine circovirus type 2 (PCV2), porcine parvovirus (PPV), pseudorabies virus (PRV), and the clinical samples suspected of PEDV described above included in this study were stored in the China-UK-NYNU-RRes Joint Laboratory of Insect Biology, Nanyang Normal University.

### Total RNA extraction

Prior to RNA extraction, frozen small intestine samples (~ 20 mg) were homogenized in liquid nitrogen. Then, total RNA was extracted by using a commercial extraction kit (EasyPure Viral DNA/RNA Kit; TransGen Biotechnology, Inc., Beijing, China) according to the manufacturer’s instructions. The RNA was dissolved in DEPC-treated water for cDNA synthesis. The extracted RNAs were suspended in 100 μl of elution buffer and stored at − 80 °C until use.

### Designing primers for RT-PSR

Using the published PEDV sequences in GenBank (NCBI), the conserved genes of the virus were analyzed, specific primer pairs were designed for the RT-PSR and RT-PCR reaction based on a conserved region of the ORF3 gene using Primer Premier 5.0 software. A pair of primer (P1 and P2) for RT-PCR were also designed. The obtained primers included a forward and reverse primer (Table 2), the 5′ sequences of the PSR-S1 and PSR-S2 primers were obtained from a botanical gene to avoid nonspecific reactions with PEDV. The RT-PSR products with repeat target sequences displayed multiple banding patterns were produced due to different spiral amplification stages by the aid of simultaneous Bst DNA polymerase extension at 3′ end and strand displacement at 5′ end. [14].

**Table 2:** Primers used in the RT-PSR and RT-PCR assay for porcine epidemic diarrhea virus (PEDV) detection

Primer	Sequence	Position within PEDV ORF3^a^
PSR-1	5′-TATTATGTTGGCAGCGCGTT-3′	207–226
PSR-2	5′-TGCCGTCATAATAAGCTGCT-3′	424–405
PSR-S1	5′-AC**GAATTC**GTACATAGAAGTATAG-*TATTATGTTGGCAGCGCGTT*-3′	207–226
PSR-S2	5′-GATATGAAGATACATGCTTAAGCA-*TGCCGTCATAATAAGCTGCT*-3′	424–405
P1	5′-GTCTGCTTTTACTCCTGGCG-3′	325–344
P2	5′-CTCAACAGTTCGCAACAGCT-3′	564–545

The text shown in italics in PSR-S1 and PSR-S2 is the central sequence, which is the same in PSR-1 and PSR-2, respectively, and the bold text is the *Eco*R I restriction site

^a^The primer position is based on the sequence of the HeN/MY/2015 strain, GenBank accession number: KU641647

### Development of the RT-PSR assay

Based on our previous experimental data, the RT-PSR reaction was performed in a volume of 25 μl, containing primers for reverse transcription and the initial amplification (0.2 mM each PSR-1 and PSR-2), the forward and reverse primers for the PSR reaction (0.8 mM each PSR-S1 and PSR-S2), dNTPs (1.5 mM), 2 U of AMV reverse transcriptase (New England Biolabs, Hitchin, UK), 8 U of *Bst* DNA polymerase (New England Biolabs), 10 mM (NH4)_2_SO_4_, 50 mM KCl, 0.1% *v*/v Tween-20, dye mix (0.025 mM phenol red and 0.08 mM cresol red), 1 μl of template RNA, and nuclease free water to 25 μl. The reaction mixture was covered with mineral oil to prevent aerosol cross contamination. PSR-amplified products were observed by the naked eye and analyzed by 2% agarose gel electrophoresis.

### Determination of the optimum temperature and time for the RT-PSR assay

Total PEDV RNA was used as the template in this reaction. After reverse transcription at 42 °C for 15 min. The PSR was conducted at various temperatures (60 °C, 61 °C, 62 °C, 63 °C, 64 °C, 65 °C, and 66 °C) to determine the optimum reaction temperature. Then, the optimum time for RT-PSR was evaluated at 30, 40, 50, and 60 min.

### Sensitivity and specificity of the RT-PSR assay

The analytical sensitivity of the RT-PSR was determined by its ability to detect a low concentration of PEDV and therefore expressed as a concentration (ng/assay) [26]. Ten-fold serial dilutions of PEDV total RNA in nuclease free water (diluted from 10^− 1^ to 10^− 6^; minimum concentration, 0.1 ng/ml) were used to calculate the sensitivity of the newly developed RT-PSR assay and compare it to that of the RT-PCR assay. The products were analyzed by separation via electrophoresis on a 2% agarose gel and directly visualized with a colorimetric pH indicator dye.

To determine the specificity of the RT-PSR assay, RNA or DNA samples from different porcine viruses, including CSFV, PRRSV, TGEV, PRV, PCV2, and PPV were tested in the assay, and ddH_2_O was used as a negative control. All viruses, except PEDV, are reference swine viruses.

The specificity of the PEDV RT-PSR assay was further evaluated by enzyme digestion of amplified products. The primers were designed with *Eco*R I restriction sites, and digestion of the amplified products should yield fragments of the expected sizes by agarose gel electrophoresis.

### Clinical sample testing

The newly developed RT-PSR assay was further evaluated using 65 clinical samples obtained from pigs with diarrhea located in Jiangxi, Jiangsu, Hunan, Hubei, and Anhui, China. Both the new RT-PSR and conventional RT-PCR assays were performed to determine the positive rate compared with virus isolation. The analytical specificity of the assays was calculated using the following definition for specificity as the percentage of false negative samples/ the number of true positive samples [26]. All products of RT-PSR were visualized after separation by electrophoresis on a 2% agarose gel. The other prevalent porcine viruses were also investigated.

## Abbreviations

CSFV: Classical swine fever virus
LAMP: Loop-mediated isothermal amplification
PCR: Polymerase chain reaction
PCV2: Porcine circovirus type 2
PEDV: Porcine epidemic diarrhea virus
PPV: Porcine parvovirus
PRRSV: Porcine reproductive and respiratory syndrome virus
PRV: Pseudorabies virus
RPA: Recombinase polymerase amplification
RT-PSR: Reverse transcription polymerase spiral reaction
TGEV: Transmissible gastroenteritis virus
